# Ozone Activates the Nrf2 Pathway and Improves Preservation of Explanted Adipose Tissue In Vitro

**DOI:** 10.3390/antiox9100989

**Published:** 2020-10-14

**Authors:** Barbara Cisterna, Manuela Costanzo, Alice Nodari, Mirco Galiè, Serena Zanzoni, Paolo Bernardi, Viviana Covi, Gabriele Tabaracci, Manuela Malatesta

**Affiliations:** 1 Department of Neurosciences, Biomedicine and Movement Sciences, Anatomy and Histology Section, University of Verona, Strada Le Grazie 8, I-37134 Verona, Italy; barbara.cisterna@univr.it (B.C.); manuela.costanzo@univr.it (M.C.); alice.nodari@univr.it (A.N.); mirco.galie@univr.it (M.G.); paolo.bernardi@univr.it (P.B.); 2 Centre for Technological Platforms, University of Verona, Piazzale L.A. Scuro 10, I-37134 Verona, Italy; serena.zanzoni@univr.it; 3 San Rocco Clinic, Via Monsignor G.V. Moreni 95, I-25018 Montichiari (BS), Italy; v.covi@brixiamed.it (V.C.); tabaracci@sanrocco.net (G.T.)

**Keywords:** fat, lipid loss, ozone therapy, scanning electron microscopy, transmission electron microscopy, real-time polymerase chain reaction, nuclear magnetic resonance spectroscopy

## Abstract

In clinical practice, administration of low ozone (O_3_) dosages is a complementary therapy for many diseases, due to the capability of O_3_ to elicit an antioxidant response through the Nuclear Factor Erythroid 2-Related Factor 2 (Nrf2)-dependent pathway. Nrf2 is also involved in the adipogenic differentiation of mesenchymal stem cells, and low O_3_ concentrations have been shown to stimulate lipid accumulation in human adipose-derived adult stem cells in vitro. Thus, O_3_ treatment is a promising procedure to improve the survival of explanted adipose tissue, whose reabsorption after fat grafting is a major problem in regenerative medicine. In this context, we carried out a pilot study to explore the potential of mild O_3_ treatment in preserving explanted murine adipose tissue in vitro. Scanning and transmission electron microscopy, Western blot, real-time polymerase chain reaction and nuclear magnetic resonance spectroscopy were used. Exposure to low O_3_ concentrations down in the degradation of the explanted adipose tissue and induced a concomitant increase in the protein abundance of Nrf2 and in the expression of its target gene Hmox1. These findings provide a promising background for further studies aimed at the clinical application of O_3_ as an adjuvant treatment to improve fat engraftment.

## 1. Introduction

During the last decades, low dosages of ozone (O_3_), a highly unstable gas that rapidly decomposes to oxygen, have increasingly been applied in O_2_-O_3_ mixtures as a successful adjuvant/complementary treatment for several diseases [1,2,3]. The therapeutic effect of low O_3_ concentrations relies in the cascade of metabolic events triggered by an initially induced mild oxidative stress, which is able to activate the antioxidant cell response but is insufficient to cause damage [4,5]; this is consistent with the principle of hormesis, i.e., “the beneficial effect of a low level exposure to an agent that is harmful at high levels” [6]. Recently, we have provided mechanistic evidence that low O_3_ concentrations stimulate in a dose-dependent manner a cytoprotective response via the Nuclear Factor Erythroid 2-Related Factor 2 (Nrf2)-mediated Keap1-dependent pathway [7] through the transcription of genes induced by antioxidant response elements (AREs).

Nrf2 also plays a regulatory role in the adipogenic differentiation of mesenchymal stem cells [8,9]. Consistent with this, we showed that treatment with low O_3_ concentrations stimulates lipid accumulation in human adipose-derived adult stem cells without altering the adipogenic process [10].

Autologous fat transplantation is nowadays commonly used in reconstructive surgery, especially for partial or total breast reconstruction in cancer patients [11], since it allows avoiding the use of silicon prosthesis implants [12]. However, fat graft survival is often suboptimal due to the progressive loss of adipocytes and the conversion of the graft into fibrous tissue and cysts [13], thus requiring multiple grafting sessions to reach optimal reconstruction [14]. New strategies to improve the viability of the harvested fat cells and graft preservation are definitely crucial [15], and O_3_ treatment may be an interesting candidate.

In this view, we carried out a pilot study using explanted mouse visceral AT maintained under in vitro conditions as a standardized experimental model, and we investigated the effects of the exposure to low O_3_ concentrations by a multimodal approach including scanning (SEM) and transmission (TEM) electron microscopy, biomolecular analyses and Nuclear Magnetic Resonance spectroscopy (^1^H NMR).

## 2. Materials and Methods

Perigonadal visceral AT was explanted from seven healthy 3-month-old female Balb/c mice used in the frame of a research project approved by the Italian Ministry of Health (ref. 538/2015-PR). Animals were handled according to the regulations of the Italian Ministry of Health (DL 4 March 2014, n. 26, directive implementation 2010/63/UE) and to the directives of the European Council (Directive 63/2010/EU of the European Parliament and of the Council). A murine model was selected because it ensures the standardized experimental conditions necessary to obtain reliable results when investigating basic biological mechanisms, and the perigonadal fat pads were chosen as typically the most accessible and abundant fat pads in the mouse. The mice were deeply anaesthetized using Tribromoethanol (TBE) and then euthanized by cervical dislocation.

The perigonadal AT was excised, cut in small pieces (1–2 mm^3^, as suggested by [16]) and washed twice with sterile phosphate buffered saline (PBS) to remove cell debris. After removing visible blood clots, the specimens were weighed and placed in culture medium (M199 containing 1% penicillin/streptomycin, 1% glutamine, 50 µg/mL gentamicin, 0.1% insulin, 1% dexamethasone) keeping a proportion of approximately 30 mg/mL [17]. Before treatment, AT pieces were maintained for 48 h in an incubator (saturating humidity, 5% CO_2_, 37 °C), and then exposed to O_2_ or O_2_-O_3_ gas mixtures (O_3_ concentrations of 10, 20 or 100 µg O_3_/mL O_2_), adapting the protocol previously set up for cell cultures [18]. Briefly, 90 mg of AT fragments were suspended in 3 mL of culture medium in a 10 mL polypropylene (O_3_ resistant) syringe (Terumo Medical Corporation, Somerset, NJ, USA); an equal volume of gas was drawn into the syringe, which was gently shaken for 10 min to facilitate the mixing of medium with gas. It has been established that a 10 min treatment allows the cell sample to react with the O_3_ dose totally [19]. The concentrations of 10 and 20 µg O_3_ were chosen as they are usually administered in clinical practice and have been shown to be non-cytotoxic for cultured adipose stem cells [10], while 100 µg O_3_ was used as a highly oxidizing condition. The gas was produced by an OZO2 FUTURA apparatus (Alnitec s.r.l., Cremosano, CR, Italy), which generates O_3_ from medical-grade O_2_, and allows photometric real-time control of gas flow rate and O_3_ concentration. The treatment with pure O_2_ was performed in order to discriminate the effect of O_3_ from O_2_ in the context of the O_2_-O_3_ gas mixtures. Controls consisted in AT samples submitted to the same handling but without exposure to O_2_ or O_2_-O_3_ gas.

After gas treatment, AT samples were placed into fresh medium in plastic dishes and maintained in the incubator until analysis. The effects were evaluated at increasing incubation times after treatment (see below). AT samples were processed for SEM and TEM, as well as for Western blot and real-time quantitative polymerase chain reaction (RT-qPCR), while the medium was collected for lactate dehydrogenase (LDH) assay and composition analysis (^1^H NMR).

### 2.1. LDH Assay

LDH, a cytosolic enzyme released by lysed cells, was evaluated as an estimate of the cytotoxic effect of gas exposure by using the CytoTox96 nonradioactive assay (Promega, Milano, MI, Italy). After treatment, AT samples were placed in the medium and LDH was measured at 2 h, 24 h and 48 h. At each time point, aliquots of medium for each condition were collected, placed in a 96 multi-well plate, mixed with Cytotox96 reagent and incubated for 30 min at room temperature. After addition of the stop solution, the absorbance was measured at 492 nm, and the data were corrected for culture medium background and normalized to the maximum LDH release (i.e., lysed sample). The results of three distinct experiments (*n* = 3) are presented as mean of percent LDH release ± standard error (SE).

### 2.2. Scanning Electron Microscopy

At 2 h, 24 h and 48 h post-treatment, AT samples were collected and immediately fixed for 2 h at 4 °C with 2% glutaraldehyde in 0.1 M phosphate buffer (PB), post-fixed with 1% OsO_4_ in the same buffer for 1 h at 4 °C, and dehydrated in acetone. Then, they were critical-point-dried (CPD 030, Balzers, Vaduz, Liechtenstein), fixed to stubs with colloidal silver, sputtered with gold by a MED 010 coater (Balzers), and examined with a XL30 SEM (FEI Company, Eindhoven, Netherlands). At least three AT samples per animal were analysed.

### 2.3. Transmission Electron Microscopy

At 2 h, 24 h and 48 h post-treatment, AT samples were collected and fixed with a 2.5% glutaraldehyde and 2% paraformaldehyde in PBS for 2 h at 4 °C. The samples were then rinsed with PBS, post-fixed with 1% OsO_4_ and 1.5% K_4_Fe(CN)_6_ for 2 h at 4 °C, dehydrated in acetone and embedded in Epon resin. Ultrathin sections were stained with Reynolds lead citrate and observed in a Philips Morgagni TEM (FEI Company) equipped with a Megaview III camera. At least three AT samples per animal were analysed.

The area of small lipid droplets extruding from the central one was measured in a total of 500 µm^2^ of cytoplasm per experimental condition (X7′100) using the ImageJ software (NIH), and their total area was calculated and expressed as percentage of the measured cytoplasmic area.

A morphometric analysis was carried out also on 30 randomly-chosen mitochondria (X28′000) per control, O_2_-, 10 µg- or 20 µg O_3_-treated samples: the mitochondrial area and the ratio between inner and outer membrane (estimating the extension of cristae independently of the mitochondrial size) were assessed. The means ± SE were calculated and a statistical comparison was performed as described below.

### 2.4. Western Blot Analysis

AT samples were collected at different post-treatment times (2 h for Nrf2; 24 h for heme oxygenase-1 (Ho-1, encoded by *Hmox1*); 24 h and 48 h for mitochondrial heat-shock protein 70, mtHsp70) and immediately frozen in liquid nitrogen to be then placed at −80 °C. Proteins were extracted according to standard procedures in RIPA buffer (150 mM NaCl, 10 mM Tris pH7.5, 1% NP40, 1% Decoxycholate, 0.1% SDS) supplemented with phosphatase and protease inhibitors (Sigma-Aldrich, Milan, MI, Italy). Samples were resolved on Tris-glycine 4–20% gradient SDS-PAGE (BIO-RAD, Segrate, MI, Italy), blotted on PVDF membrane (BIO-RAD), and developed with ECL Western Blotting Substrate (Thermo Scientific, Rodano, MI, Italy). The following antibodies were used: anti-Nrf2 1:1000 (ab62532 Abcam, Cambridge, United Kingdom), anti-Ho-1 1:500 (3391-100 BioVision, Inc), anti-mtHsp70 1:1000 (ALX-804-077-R100, Enzo Life Sciences, Farmingdale, NY, USA), anti-Gapdh 1:5000 (ab181602, Abcam) and βActin 1:5000 (ab8226 Abcam).

### 2.5. Real-Time Quantitative Polymerase Chain Reaction

At 4 h post-treatment, AT samples were collected and RNA was extracted using the Qiagen RNAeasy Plus mini kit (ref. 74134). cDNA was generated by SuperScript™ III Reverse Transcriptase (Invitrogen, Carlsbad, CA, USA; cat. no. 18080093) and used with Applied Biosystems SYBR™ Green PCR Master Mix (Applied Biosystems, Foster City, CA, USA; cat. no. 4309155) for real-time qPCR analysis (Hmox1 forward primer: AGGTACACATCCAAGCCGAGA, Hmox1 reverse primer: CATCACCAGCTTAAAGCCTTCT). Assays were performed using an Applied Biosystems Step-One Real-Time PCR System.

### 2.6. Nuclear Magnetic Resonance Spectroscopy

The identification and quantification of AT metabolites released in the culture medium were performed by ^1^H NMR.

Because AT samples treated with 100 µg O_3_ were found to be necrotic already 2 h post-treatment, they were excluded from the metabolomics analysis.

One-ml aliquots of culture medium per each experimental condition were collected at 2 h, 24 h and 48 h post-treatment, immediately frozen in liquid nitrogen and placed at −80 °C. Experiments were performed with a Bruker Avance III 600 MHz spectrometer equipped with a TCI cryoprobe operating at 298 K. The samples were defrosted on ice and 500 μL were mixed with 50 μL of D_2_O containing TSP, as reference. The ^1^H NMR spectra of the culture media were obtained by the water-suppressed standard 1D Carr-Purcell-Meiboom-Gill pulse sequence. The free induction decays (FIDs) were recorded by 32K data points with a spectral width of 7200 Hz and 64 scans with a relaxation delay of 4.0 s.

For data processing and multivariate analysis, see Supplementary Materials, Multivariate Data Analysis, and Methods.

### 2.7. Statistical Analysis

Statistical analysis of the LDH assay, morphometric evaluation of lipid droplets and mitochondria and RT-qPCR was performed by the one-way ANOVA, followed by Tukey’s pairwise test of the. For Western blots, linear regression modelling was used to test the dose-dependence hypothesis. In the case of mtHsp70, one-way ANOVA was performed, followed by Dunnett’s multiple comparisons test to examine for significant differences with the control. Statistical difference in the mean value of selected metabolites was assessed using one-way ANOVA followed by Tukey’s pairwise test. For all statistical tests, significant difference was set at *p* ≤ 0.05.

## 3. Results and Discussion

### 3.1. LDH Assay

**Figure 1:**
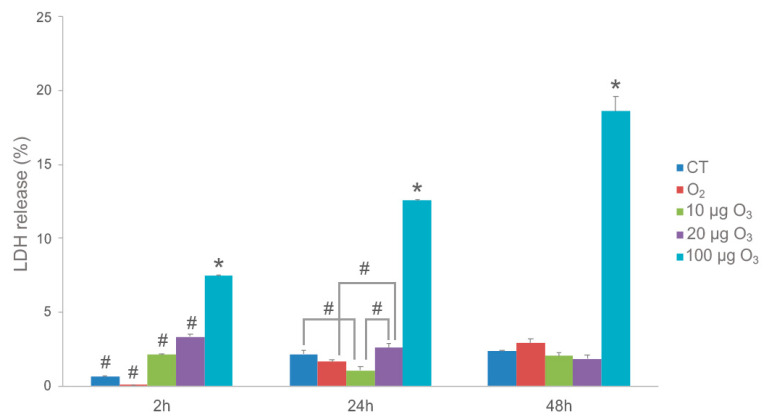
LDH assay on AT samples. Histograms show the mean value ± SE of percentage of tissue viability after 2 h, 24 h and 48 h from the treatment. Statistical difference is indicated by # (*p* < 0.05). The value referring to 100 μg O_3_ is statistically different from all other samples at each time point (* *p* < 0.001). CT, control.

Two hours post-treatment, cell death (as estimated by LDH release) in the AT samples was slightly increased after O_2_-O_3_ gas exposure at all O_3_ concentrations in comparison with the control and O_2_-treated samples (Figure 1). Although the difference was statistically significant, the LDH values were always below 4% for both 10 and 20 μg O_3_, reaching about 8% in samples treated with 100 μg O_3_.

The effect of this initial stress decreased at later time points: in fact, after 24 h, AT treated with 10 μg O_3_ showed even lower LDH values in comparison to control and 20 μg O_3_-treated samples, suggesting that, in our experimental model, the concentration of 10 μg O_3_ may be optimal to induce a cytoprotective mechanism. Conversely, LDH values in response to 100 μg O_3_ remained the highest. After 48 h, no significant difference in LDH release was found among control, O_2_-, 10 μg O_3_- and 20 μg O_3_-treated samples, whereas 100 μg O_3_ yielded significantly higher values, probably due to strong oxidative stress.

In summary, these data demonstrate that 10 and 20 μg O_3_ treatments do not induce appreciable cell death in explanted AT, which is consistent with previous findings in cultured cells [10,18,20].

### 3.2. Scanning and Transmission Electron Microscopy

Ultrastructural analysis by SEM and TEM confirmed that the protocol we used [16] allows good preservation of explanted AT in vitro.

At SEM, all AT samples were typically characterized by unilocular mature adipocytes, surrounded by an extracellular scaffolding network of thin collagen fibres.

**Figure 2:**
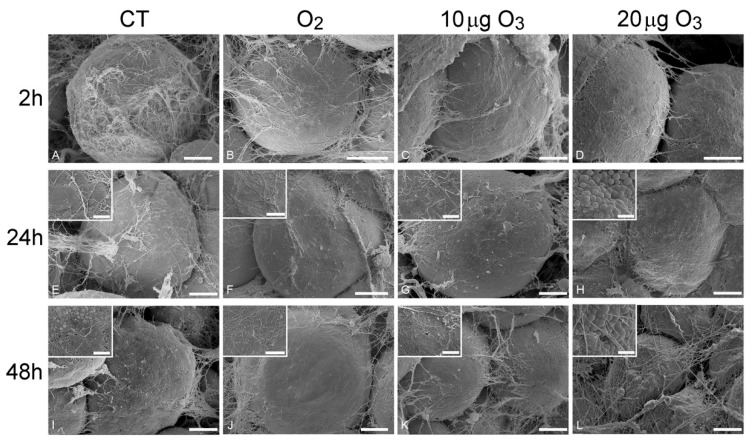
Scanning electron micrographs of adipocytes 2 h (**A**–**D**), 24 h (**E**–**H**) and 48 h (**I**–**L**) after gas treatment. At 2 h, the adipocytes were spherical with smooth surface and surrounded by an extracellular network of thin collagen fibres in control (**A**), O_2_- (**B**), 10 µg O_3_- (**C**) and 20 µg O_3_- (**D**) treated samples. At 24 h, control (**E**), O_2_- (**F**) and 10 µg O_3_- (**G**) treated samples maintained a well-preserved morphology, while adipocytes exposed to 20 µg O_3_ (**H**) showed small lipid droplets budding from the surface. At 48 h, control adipocytes (**I**) showed clusters of lipid droplets budding from the surface; O_2_ (**J**) and 10 µg O_3_ (**K**)-treated samples showed well-preserved spherical adipocytes with smooth surface. 20 µg O_3_-treated samples (**L**) showed slight depressions and many budding lipid droplets. CT, control. Bars, 10 µm (**A**–**L**), 2 µm (insets).

At 2 h post-treatment, AT exposed to either O_2_ (Figure 2B), 10 μg O_3_ (Figure 2C) or 20 μg O_3_ (Figure 2D) showed spherical adipocytes with a smooth surface, similar to control (Figure 2A); conversely, adipocytes exposed to 100 μg O_3_ were wrinkled (Figure S1A).

At 24 h, AT samples treated with O_2_ (Figure 2F) and 10 μg O_3_ (Figure 2G) maintained a well-preserved morphology comparable to control (Figure 2E), whereas the adipocytes of samples treated with 20 μg O_3_ (Figure 2H) showed a few small lipid droplets budding from their surface; samples exposed to 100 μg O_3_ were characterized by markedly wrinkled adipocytes (not shown).

After 48 h, in control (Figure 2I) and 20 μg O_3_-treated samples (Figure 2L), several adipocytes showed clusters of lipid droplets budding from their surface, while samples treated with O_2_ (Figure 2J) and 10 μg O_3_ (Figure 2K) showed spherical adipocytes with smooth surface; in samples treated with 100 μg O_3_, adipocytes were shrunken, with evident plasma membrane breakages (not shown).

Previous studies on human AT harvested by liposuction demonstrated that chemical and mechanical stress triggers an active process of lipid loss in adipocytes [21]: depending on the stress intensity, the lipid loss may result in the extrusion of a few small droplets (through the micropores that transitorily form in the plasmalemma, while cell structure and viability are preserved) or the adipocyte may become wrinkled due to a massive release of large droplets, until depleted adipocytes acquire a cup-like morphology. Consistently, in the present study, AT samples treated with 100 μg O_3_ underwent rapid and massive lipid loss, a concentration of 20 μg O_3_ induced lipid budding from the cell surface thus indicating a mild stress, while O_2_ and 10 μg O_3_ did not cause any lipid loss, even 48 h after gas treatment.

**Figure 3:**
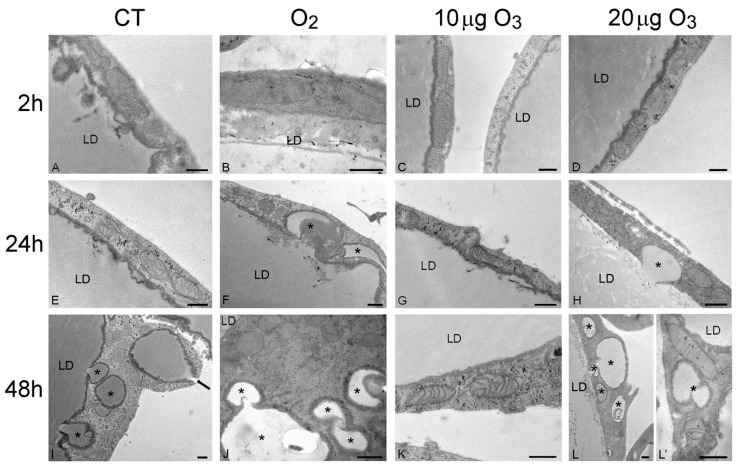
Transmission electron micrographs of the peripheral cytoplasmic rim of adipocytes 2 h (**A**–**D**), 24 h (**E**–**H**) and 48 h (**I**–**L**) after gas treatment. At 2 h, all cytoplasmic organelles were well preserved in control (**A**), O_2_- (**B**), 10 µg O_3_- (**C**) and 20 µg O_3_- (**D**) treated samples. At 24 h, control (**E**) and 10 µg O_3_- (**G**) treated samples maintained a good morphology, while adipocytes exposed to O_2_ (**F**) or 20 µg O_3_ (**H**) showed small lipid droplets (asterisks) budding from the large central one. At 48 h, control (**I**), O_2_- (**J**) and 20 µg O_3_ (**L**,**L′**)-treated samples showed many lipid droplets (asterisks) budding from the central one or distributed in the cytoplasm; note (**I**) the lipid droplet extruding from the cell (arrow). O_2_- (**J**) and 20 µg O_3_ (**L′**)-treated adipocytes also showed mitochondria poor in cristae. Conversely, 10 µg O_3_-treated adipocytes (**K**) showed excellent structural preservation. CT, control; LD, central lipid droplet. Bars, 500 nm.

The SEM findings were corroborated and extended by the TEM observations. At 2 h post-treatment, control (Figure 3A), O_2_- (Figure 3B), 10 μg O_3_- (Figure 3C) and 20 μg O_3_-treated samples (Figure 3D) showed well-preserved unilocular adipocytes surrounded by a thin cytoplasmic sheet where organelles and a flat nucleus were located; mitochondria were numerous, elongated and rich in cristae; the tubules and vesicles of the smooth endoplasmic reticulum were particularly abundant; the rough endoplasmic reticulum and Golgi complexes were well developed; and many glycogen granules were distributed in the cytosol. Following 100 μg O_3_, the adipocytes underwent evident necrosis, with marked lipid loss and barely recognizable organelles (Figure S1B).

After 24 h, adipocytes of control (Figure 3E) and 10 μg O_3_-treated samples (Figure 3G) were similar to those at 2 h post-treatment; after exposure to O_2_ (Figure 3F) or 20 μg O_3_ (Figure 3H), lipid droplets were observed budding from the main droplet while all the other organelles were well preserved. The adipocytes treated with 100 μg O_3_ were completely necrotic (not shown).

After 48 h, in control samples (Figure 3I) the adipocytes showed several small lipid droplets distributed in the cytoplasm and some of them were found to be extruding from the cell; however, no alteration of cell organelles was observed. In samples exposed to 10 μg O_3_ (Figure 3K), no lipid extrusion or organelle modification was ever observed, adipocyte morphology being very similar as at 2 h post-treatment. In samples treated with O_2_ (Figure 3J) or 20 μg O_3_ (Figure 3L,L′) many lipid droplets were found budding from the large central droplet and scattered in the cytoplasm; moreover, most mitochondria showed a few cristae, although all other organelles were still well preserved.

Consistent with the morphological observations, morphometric evaluation (Table 1) demonstrated that, after 24 h, the amount of small lipid droplets scattered in the cytoplasm was significantly higher in O_2_- and 20 μg O_3_-treated samples in comparison to control, while 10 μg O_3_-treated samples showed values similar to control. After 48 h, control, O_2_- and 20 μg O_3_-treated samples contained similar large amounts of lipid droplets distributed in the cytoplasm, whereas in 10 μg O_3_-treated adipocytes the value remained markedly lower, thus supporting the cytoprotective effect of this O_3_ concentration.

The presence of lipid droplets in the peripheral cytoplasmic rim is one of the typical features at the onset of adipocyte apoptosis [22]; however, in our AT samples, we never observed apoptotic signs, such as nuclear chromatin condensation, hypertrophic mitochondria, or dilated endoplasmic reticulum. This suggests that the lipid loss observed in our samples may represent an active response to cell stress aimed at preserving cell viability, as hypothesized by Conti et al. [21]. Accordingly, under another stressing condition (nanoparticle-mediated hyperthermia [23]), rapid lipid loss was observed in adipocytes that were fully vital with unchanged ultrastructural features.

Treatment with O_2_, 10 and 20 μg O_3_, proved to be non-toxic even after 48 h, as damaged organelles were never observed; on the contrary, the highly oxidizing concentration of 100 μg O_3_ induced rapid and massive necrosis. However, morphometric evaluation demonstrated that, while no mitochondrial size alteration occurred (Table 2), a significant reduction in the length of mitochondrial cristae took place in adipocytes 48 h post-treatment with O_2_ and 20 μg O_3_ (Table 3).

This is suggestive of reduced and/or altered respiratory capability [24]. It is known that mitochondrial functions are strictly related to the level of reactive oxygen species (review in [25]), and it is likely that the oxidative stress due to O_2_ and 20 μg O_3_ (although unable to cause organelle damage) could induce functional alteration at the longest incubation time. In contrast, 10 μg O_3_ proved to be safe, consistent with previous demonstration that appropriate O_3_ concentrations may even positively affect mitochondrial activity [26,27].

### 3.3. Biomolecular Analyses

Nrf2 belongs to the cap‘n’collar basic-region (CNC) leucine zipper transcription factor family together with NF-E2, Nrf1 and Nrf3; it plays a primary role in the cellular response to O_3_ exposure and, more generally, to oxidative stress, being involved in multiple metabolic pathways [28].

**Figure 4:**
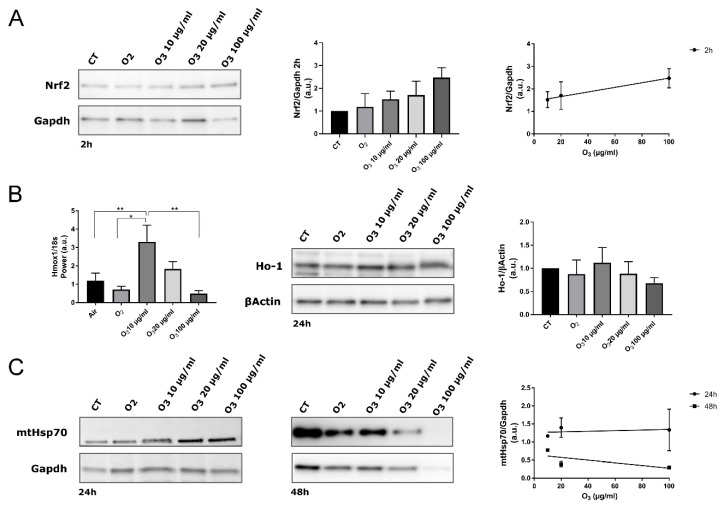
Biomolecular analysis of Nrf2, Hmox1/Ho-1 and mtHsp70 in control (CT), O_2_-, 10 µg O_3_-, 20 µg O_3_- and 100 µg O_3_-treated AT samples. (**A**) Western blot of Nrf2 protein. Nrf2 stabilization was observed 2 h after O_3_ treatment (Western blot and histogram). The protein level is proportional to the O_3_ concentration (linear regression *p* = 0.05; right). (**B**) Gene expression of Hmox-1 in AT at 4 h post-treatment (* *p* < 0.05, ** *p* < 0.005; left). The maximum level of Hmox1 expression was reached by treating samples with 10 µg/mL O_3_. The amount of Ho-1 protein was evaluated at 24 h post-treatment (Western blot and histogram; right). (**C**) mtHsp70 protein was evaluated at 24 h and 48 h post-treatment (left). The protein level is not significantly proportional to the O_3_ concentration (linear regression 24 h: *p* = 0.8; 48 h: *p* = 0.5; right). The values presented are means ± SE of 3 independent experiments. Data were normalized to the level of housekeeping proteins (Gapdh and βActin) and expressed as in proportion to the levels in control samples.

In our AT samples, 2 h post-treatment, the total amount of Nrf2 protein in all O_3_-treated tissue increased in proportion to the O_3_ concentration, as demonstrated by the linear regression analysis (Figure 4A).

It has been already demonstrated in different cell types [7,20,29] that mild O_3_ treatment leads to a specific antioxidant response by inducing gene transcription via AREs. By combining sub-nuclear tracking of Nrf2 localization at fluorescence and electron microscopy and functional genetic engineering approaches, it was demonstrated that low O_3_ concentrations such as 10 and 20 μg increase Nrf2 protein stability by preventing its Keap1-mediated degradation [7,30]. In fact, even under a mild oxidative stress due to low O_3_ concentrations, Nrf2 rapidly dissociates from its negative regulator Keap1 and translocates into the nucleus, thus activating the expression of ARE-driven genes [7,30]. This regulatory mechanism is very efficient, since it ensures the rapid transcription of antioxidant genes without requiring the de novo synthesis of Nrf2. Moreover, a combined approach of microarray gene expression and real-time qPCR with ultrastructural immunocytochemistry allowed identifying the antioxidant genes up-regulated through Nrf2 activation in response to O_2_-O_3_ gas mixtures used for O_3_ therapy [20]. In particular, concentrations of 10 and 16 μg O_3_ were found to induce genes involved in the cellular response to stress (Hmox1, ERCC4, CDKN1A) and in the transcription machinery (CTDSP1). Finally, a recent microarray analysis of gene expression correlated the expression of Hmox1 gene with the therapeutic use of O_3_ in myocardial ischemia/reperfusion injury [29]. It is therefore well established that the O_3_-mediated activation of Nrf2 induces the transcription of antioxidant genes. Accordingly, RT-qPCR demonstrated a statistically significant upregulation of *Hmox1*, a well-known Nrf2 target gene whose expression is usually increased after Nrf2 activation [31], in explanted AT treated with 10 μg O_3_ in comparison with CT, O_2_-, and 100 μg O_3_-treated samples (Figure 4B). On the other hand, AT samples treated with 20 μg O_3_ showed an increased though not significant activation of the *Hmox1* gene. Consistently, the expression of Ho-1, which is encoded by *Hmox1* and is involved in many cytoprotective pathways exerting anti-oxidant, anti-inflammatory and anti-apoptotic effects [32], showed the same pattern as *Hmox1* gene expression, albeit without statistically significant differences among the experimental conditions. It may be therefore hypothesized that Hmox1 upregulation contributes to the preservation of explanted AT treated with 10 μg O_3_.

Interestingly, according to previous evidence [7,20], in AT samples pure O_2_ negligibly affected Nrf2 activation, further supporting the cytoprotective role of O_3_.

It is known that ARE gene expression can also be induced by Nrf1, which plays multiple roles but distinct from those of Nrf2 [33,34]. However, to our knowledge, no evidence on the involvement of Nrf1 in the cytoprotective response induced by mild O_3_ treatment has been reported so far. Increased expression of Nrf1 has been found only in mice exposed to repeated inhalation of high doses of O_3_ [35], probably due to its role in modulating the inflammatory response [33]. Likewise, no data are available on the involvement of Nrf3 in the cell response to O_3_; this factor, which has been poorly studied in comparison to the other members of the CNC family, seems to play a role in differentiation, inflammation, and carcinogenesis [36], and can negatively regulate ARE-mediated gene expression [37]. Future investigations into the mechanisms accounting for the cytoprotective response following mild O_3_ exposure should thus also take into consideration the possible contributions of Nrf1 and Nrf3 besides that of Nrf2.

Among its various functions, Nrf2 is involved in mitochondrial biogenesis [38,39]; moreover, mitochondrial respiration and ATP synthesis are strictly related to Nrf2/Keap1 levels, which play a crucial role in the functional modulation of mitochondria under stress conditions in order to preserve cell redox homeostasis [40,41,42]. The O_3_-induced activation of Nrf2 may therefore contribute to the excellent preservation of mitochondria in 10 μg O_3_-treated AT, in comparison to AT treated with O_2_ (which induced a lower level of Nrf2 activation), further supporting the conclusion that, in our experimental conditions, this O_3_ concentration is able to yield an optimal balance between oxidative stress and antioxidant response.

The expression of mtHsp70 also correlates with mitochondrial biogenesis and activity, being consequently central for cell survival; in fact, its expression is induced by various mitochondrial stresses [43] or by common inducers of the Keap1/Nrf2/ARE pathway, but independently of Nrf2 [44]. As shown by linear regression analysis, the amount of mtHsp70 did not correlate with O_3_ concentration at any time point (Figure 4C). After 48 h, ANOVA revealed a significantly reduced amount of mtHsp70 in all treated AT samples compared with control (O_2_: *p* = 0.0007; 10 μg O_3_: *p* = 0.0185; 20 μg O_3_: *p* = 0.0002; 100 μg O_3_: *p* = 0.0001). Interestingly, this reduction was particularly marked in samples treated with O_2_, 20 μg O_3_ and 100 μg O_3_, suggesting that mtHsp70 failed to preserve mitochondrial integrity at later time points. This finding is consistent with the morphological and morphometric analyses by TEM that provided evidence of mitochondrial alteration (in O_2_- and 20 μg O_3_-treated AT samples) or damage (in 100 μg O_3_-treated samples), whereas mitochondria in 10 μg O_3_-treated samples were well preserved.

### 3.4. Nuclear Magnetic Resonance Spectroscopy

By this method, we performed a metabolomic analysis to identify specific metabolites released in the medium that reflect the functional response of AT to in vitro culture and treatment.

**Figure 5:**
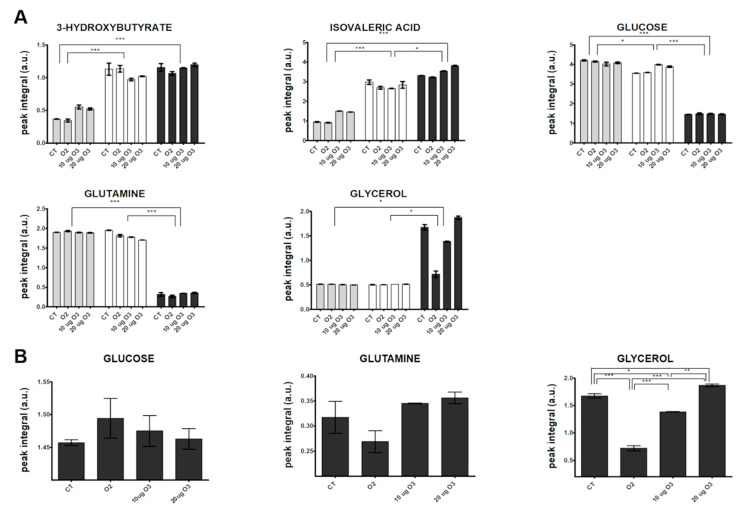
Changes in the relative concentration (arbitrary NMR integral units, mean values ± SE, *n* = 2) of selected metabolites in the media at different incubation times post-treatment of AT culture (2 h: grey; 24 h: white; 48 h: dark grey) (**A**) and among the different samples at 48 h (**B**). CT, control. Significant statistical differences are indicated as *** *p* ≤ 0.001, ** *p* ≤ 0.01 and * *p* ≤ 0.05.

Visual inspection of the 1D ^1^H NMR spectra recorded on culture media derived from AT samples after gas exposure showed that the peak intensity and position of several metabolites were different among the samples (representative spectra are shown in Supplementary Materials, Figure S2). The variability and complexity of the spectra were interpreted by a multivariate statistical approach to identify the meaningful metabolites discriminating treated and control samples (Supplementary Materials, Multivariate data analysis, Results, Figure S3). Figure 5A shows the trend along the incubation times post-treatment of the identified metabolites with unequivocal assignment obtained from NMR (coefficients of variation for each analysed metabolite are reported in Supplementary Materials, Table S1). In the culture media, the levels of most metabolites remained constant at 2 h and 24 h. Only the levels of 3-hydroxybutyrate and isovaleric acid increased 24 h post-treatment in all samples, suggesting an increase in fatty acid catabolism. The 3-hydroxybutyrate is synthesized from acetyl-CoA through β-oxidation of fatty acids [45], and it is considered a signalling metabolite because it is taken up by peripheral tissues and oxidized for ATP production [46,47]. This finding therefore suggests that some lipid catabolism occurs in all AT samples after 24 h in culture independent of treatment, probably as a consequence of maintenance under in vitro conditions.

However, the strongest variations were observed at 48 h, when a marked decrease in the relative content of two main carbon sources (glucose and glutamine) and an increase in glycerol and lactate were observed in all samples. The enhanced uptake of glucose and the release of free glycerol and lactate in the media from 24 to 48 h post-treatment indicate an active synthesis of glycerol from glucose and an accelerated triacylglycerol turnover. A massive efflux of free glycerol has been reported in white AT with low lipogenic activity and accelerated triacylglycerol turnover, where most fatty acids were recycled by mature adipocytes [48]. The decreased glutamine level observed at 48 h may be explained by its active transport into cells, where it is converted to glutamate that in turn enters the tricarboxylic acid (TCA) cycle.

In order to evaluate the effect of O_3_ treatment on explanted AT, a targeted inspection of metabolite levels was performed at 48 h (Figure 5B). The glucose level was higher in the medium of O_3_-treated samples as compared with the control. In the presence of oxidative stress, adipocytes demonstrate a lower uptake of glucose due to translocation of glucose transporter type 4 and decreased activity of phosphatidylinositol 3 kinase [49]. A similar effect has been reported by Saleh et al. [50] in rats systemically administered O_3_. The marked decrease of free glycerol in the medium after treatment with O_2_ or 10 μg O_3_ suggests that the low level of oxidative stress slows down glycerol efflux from adipocytes. In particular, a key role in limiting glycerol loss could be played by the O_3_-induced pro-adipogenic activity of Nrf2 [8,9,51,52,53,54]. In contrast, the same samples showed decreased amounts of glutamine in the medium, suggesting a higher glutamine consumption rate. Glutamine uptake has been shown to be significantly enhanced to maintain TCA cycle function, in which acetyl-CoA is formed from glutamine via reductive carboxylation [55]. Low O_3_ concentrations may therefore be involved in the metabolic regulation of the TCA cycle under stress conditions. Interestingly, it has been hypothesized that a protective mechanism related to enhanced uptake of glutamine may be mediated by Hsp70 [56], a protein previously shown to be upregulated by treatment with low O_3_ concentrations both in vivo and in vitro [18,57].

## 4. Conclusions

The multimodal approach used in the present study to monitor explanted AT maintained in vitro showed that progressive functional and structural alterations occur in adipocytes, such as increased lipid catabolism, low lipogenic activity and, finally, lipid loss. However, in our experimental model, exposure to low O_3_ concentrations administered as O_2_-O_3_ gas mixtures was found to protect the explanted AT from this progressive degradation. Both 10 μg O_3_ and 20 μg O_3_—but not O_2_—were able to increase Nrf2 protein levels; moreover, 10 μg O_3_ induced also a significant upregulation of *Hmox1*, belonging to the ARE-regulated genes, suggesting that the Nrf2/Keap1 cytoprotective pathway was activated. These results support the notion of the primary role of low O_3_ concentrations in the induction of the antioxidant response. Despite some cell lysis observed immediately after gas exposure, neither 10 μg O_3_ nor 20 μg O_3_ caused additional cell damage until 48 h, while inducing metabolic adaptations related to oxidative stress. It is worth noting that 10 μg O_3_ was especially effective in preserving adipocytes in comparison to 20 μg O_3_, causing a lower cell death rate immediately after exposure, maintaining excellent mitochondrial structure and preventing lipid loss until 48 h. Therefore, under our experimental conditions, the concentration of 10 μg O_3_ was found to be optimal for adipocytes in terms of cell viability, structural and functional preservation, and lipid stock maintenance. This O_3_ concentration likely induced an oxidative “eustress” [58] able to stimulate Nrf2-mediated metabolic pathways responsible for the cytoprotective response, without adverse cytological consequences. In addition, the finding that 10 μg O_3_ exerts an adipogenic effect on human adipose-derived adult stem cells [10] supports the hypothesis that the mild oxidative stress induced by this low O_3_ concentration is able to promote adipogenesis via the Nrf2/Keap1 pathway.

It is known that murine AT and human AT share several structural and functional similarities but differ in some species-specific peculiarities that characterize the visceral and subcutaneous fat pads [59]. However, it is worth noting that, to our knowledge, no study has been carried out to evaluate at the cellular level the effects of mild O_3_ treatment on AT in both rodents and humans. Therefore, this pilot work, although performed in an animal explant model, provides a solid scientific background for further studies on human AT aimed at validating the potential of mild O_3_ treatment in reconstructive medicine. Clinical studies are ultimately warranted to test the efficacy of O_3_ adjuvant treatment in improving graft survival in patients submitted to autologous fat transplantation, and to guide the selection of the most appropriate protocols for pre-implant AT treatment and/or post-graft local or systemic O_3_ administration.

## Figures and Tables

**Table 1:** Mean ± SE of percentage values of cytoplasmic area occupied by the small lipid droplets budding from the main central droplet. Asterisks indicate significant difference from control (** *p* < 0.01; *** *p* < 0.001).

	Control	O_2_	10 μg O_3_	20 μg O_3_
24 h	0.198 ± 0.142	11.742 ± 2.873 **	0.049 ± 0.034	9.024 ± 0.009 **
48 h	23.697 ± 3.518	29.992 ± 3.023	0.291 ± 0.267 ***	27.827 ± 2.479

**Table 2:** Mitochondrial area values (mean ± SE) according to treatment and incubation time post-treatment.

	Control	O_2_	10 μg O_3_	20 μg O_3_
2 h	0.119 ± 0.013	0.117 ± 0.014	0.090 ± 0.010	0.094 ± 0.012
24 h	0.093 ± 0.009	0.098 ± 0.008	0.079 ± 0.010	0.085 ± 0.009
48 h	0.089 ± 0.009	0.091 ± 0.007	0.085 ± 0.009	0.073 ± 0.007

**Table 3:** Mitochondrial inner/outer membrane ratio (mean ± SE) according to treatment and incubation time post-treatment. Asterisks indicate statistical difference from control (* *p* < 0.05).

	Control	O_2_	10 μg O_3_	20 μg O_3_
2 h	1.989 ± 0.154	2.123 ± 0.142	2.019 ± 0.135	1.965 ± 0.176
24 h	1.811 ± 0.101	1.848 ± 0.226	1.903 ± 1.104	1.710 ± 0.090
48 h	1.841 ± 0.131	1.283 ± 0.176 *	1.862 ± 0.107	1.341 ± 0.163 *

## Supplementary Materials

The following are available online at https://www.mdpi.com/2076-3921/9/10/989/s1, Figure S1. SEM (**A**) and TEM (**B**) micrographs of adipocytes after 2 h from treatment with 100 µg O_3_; Figure S2. Representative NMR spectra recorded on culture media after 2 h (green), 24 h (magenta) and 48 h (blue) from gas treatment; Figure S3. NMR-based metabolomic analysis of culture medium samples; Table S1. Coefficient of variation (CV% = standard deviation / mean × 100) for each metabolite analysed (*n* = 2).

## References

[1] Elvis A.M., Ekta J.S. (2011). Ozone therapy: A clinical review. J. Nat. Sci. Biol. Med..

[2] Bocci V. (2012). How a calculated oxidative stress can yield multiple therapeutic effects. Free Radic. Res..

[3] Viebahn-Hänsler R., Leon Fernandez O.S., Fahmy Z. (2012). Ozone in medicine: The low dose ozone concept—Guidelines and treatment strategies. Ozone Sci. Eng. J. Int. Ozone Assoc..

[4] Re L. (2008). La terapia con ossigeno-ozono o ozormesi: Recenti acquisizioni scientifiche. Med. Med..

[5] Re L., Mawsouf M.N., Menéndez S., León O.S., Sánchez G.M., Hernández F. (2008). Ozone therapy: Clinical and basic evidence of its therapeutic potential. Arch. Med. Res..

[6] Goldman M. (1996). Cancer risk of low-level exposure. Science.

[7] Galiè M., Costanzo M., Nodari A., Boschi F., Calderan L., Mannucci S., Covi V., Tabaracci G., Malatesta M. (2018). Mild ozonisation activates antioxidant cell response by the Keap1/Nrf2 dependent pathway. Free Radic. Biol. Med..

[8] Hou Y., Xue P., Bai Y., Liu D., Woods C.G., Yarborough K., Fu J., Zhang Q., Sun G., Collins S. (2012). Nuclear factor erythroid-derived factor 2-related factor 2 regulates transcription of CCAAT/enhancer-binding protein during adipogenesis. Free Radic. Biol. Med..

[9] Vomhof-DeKrey E.E., Picklo M.J. (2012). NAD(P)H:quinone oxidoreductase 1 activity reduces hypertrophy in 3T3-L1 adipocytes. Free Radic. Biol. Med..

[10] Costanzo M., Boschi F., Carton F., Conti G., Covi V., Tabaracci G., Sbarbati A., Malatesta M. (2018). Low ozone concentrations promote adipogenesis in human adipose-derived adult stem cells. Eur. J. Histochem..

[11] Coleman S.R., Saboeiro A.P. (2007). Fat grafting to the breast revisited: Safety and efficacy. Plast. Reconstr. Surg..

[12] Howes B.H., Fosh B., Watson D.I., Yip J.M., Eaton M., Smallman A., Dean N.R. (2014). Autologous fat grafting for whole breast reconstruction. Plast. Reconstr. Surg. Glob. Open.

[13] Niechajev I., Sevćuk O. (1994). Long-term results of fat transplantation: Clinical and histologic studies. Plast. Reconstr. Surg..

[14] De Decker M., De Schrijver L., Thiessen F., Tondu T., Van Goethem M., Tjalma W.A. (2016). Breast cancer and fat grafting: Efficacy, safety and complications-a systematic review. Eur. J. Obstet. Gynecol. Reprod. Biol..

[15] Peltoniemi H.H., Salmi A., Miettinen S., Mannerström B., Saariniemi K., Mikkonen R., Kuokkanen H., Herold C. (2013). Stem cell enrichment does not warrant a higher graft survival in lipofilling of the breast: A prospective comparative study. J. Plast. Reconstr. Aesthet. Surg..

[16] Carswell K.A., Lee M.J., Fried S.K. (2012). Culture of isolated human adipocytes and isolated adipose tissue. Methods Mol. Biol..

[17] Fried S.K., Moustaid-Moussa N. (2001). Culture of adipose tissue and isolated adipocytes. Methods Mol. Biol..

[18] Costanzo M., Cisterna B., Vella A., Cestari T., Covi V., Tabaracci G., Malatesta M. (2015). Low ozone concentrations stimulate cytoskeletal organization, mitochondrial activity and nuclear transcription. Eur. J. Histochem..

[19] Larini A., Bianchi L., Bocci V. (2003). The ozone tolerance: I) Enhancement of antioxidant enzymes is ozone dose-dependent in Jurkat cells. Free Radical Res..

[20] Scassellati C., Costanzo M., Cisterna B., Nodari A., Galiè M., Cattaneo A., Covi V., Tabaracci G., Bonvicini C., Malatesta M. (2017). Effects of mild ozonisation on gene expression and nuclear domains organization in vitro. Toxicol. In Vitro.

[21] Conti G., Benati D., Bernardi P., Jurga M., Rigotti G., Sbarbati A. (2014). The post-adipocytic phase of the adipose cell cycle. Tissue Cell.

[22] Murano I., Rutkowski J.M., Wang Q.A., Cho Y.R., Scherer P.E., Cinti S. (2013). Time course of histomorphological changes in adipose tissue upon acute lipoatrophy. Nutr. Metab. Cardiovasc. Dis..

[23] Marinozzi M.R., Pandolfi L., Malatesta M., Colombo M., Collico V., Lievens P.M., Tambalo S., Lasconi C., Vurro F., Boschi F. (2017). Innovative approach to safely induce controlled lipolysis by superparamagnetic iron oxide nanoparticles-mediated hyperthermic treatment. Int. J. Biochem. Cell Biol..

[24] Leveille C.F., Mikhaeil J.S., Turner K.D., Silvera S., Wilkinson J., Fajardo V.A. (2017). Mitochondrial cristae density: A dynamic entity that is critical for energy production and metabolic power in skeletal muscle. J. Physiol..

[25] Mailloux R.J., Jin X., Willmore W.G. (2014). Redox regulation of mitochondrial function with emphasis on cysteine oxidation reactions. Redox Biol..

[26] Madej P., Plewka A., Madej J.A., Plewka D., Mroczka W., Wilk K., Dobrosz Z. (2007). Ozone therapy in induced endotoxemic shock. II. The effect of ozone therapy upon selected histochemical reactions in organs of rats in endotoxemic shock. Inflammation.

[27] Lintas G., Molinari F., Simonetti V., Franzini M., Liboni W. (2013). Time and time-frequency analysis of near-infrared signals for the assessment of ozone autohemotherapy long-term effects in multiple sclerosis. Conf. Proc. IEEE Eng. Med. Biol. Soc..

[28] Galiè M., Covi V., Tabaracci G., Malatesta M. (2019). The Role of Nrf2 in the Antioxidant Cellular Response to Medical Ozone Exposure. Int. J. Mol. Sci..

[29] Jiang T., Liu Y., Chen B., Si L. (2020). Identification of potential molecular mechanisms and small molecule drugs in myocardial ischemia/reperfusion injury. Braz. J. Med. Biol. Res..

[30] Itoh K., Wakabayashi N., Katoh Y., Ishii T., O’Connor T., Yamamoto M. (2003). Keap1 regulates both cytoplasmic-nuclear shuttling and degradation of Nrf2 in response to electrophiles. Genes Cells.

[31] Kansanen E., Kuosmanen S.M., Leinonen H., Levonen A.L. (2013). The Keap1-Nrf2 pathway: Mechanisms of activation and dysregulation in cancer. Redox Biol..

[32] Loboda A., Damulewicz M., Pyza E., Jozkowicz A., Dulak J. (2016). Role of Nrf2/HO-1 system in development, oxidative stress response and diseases: An evolutionarily conserved mechanism. Cell. Mol. Life Sci..

[33] Biswas M., Chan J.Y. (2010). Role of Nrf1 in antioxidant response element-mediated gene expression and beyond. Toxicol. Appl. Pharmacol..

[34] Zhang Y., Xiang Y. (2016). Molecular and cellular basis for the unique functioning of Nrf1, an indispensable transcription factor for maintaining cell homoeostasis and organ integrity. Biochem. J..

[35] Zhong J., Allen K., Rao X., Ying Z., Braunstein Z., Kankanala S.R., Xia C., Wang X., Bramble L.A., Wagner J.G. (2016). Repeated ozone exposure exacerbates insulin resistance and activates innate immune response in genetically susceptible mice. Inhal. Toxicol..

[36] Chevillard G., Blank V. (2011). NFE2L3 (NRF3): The Cinderella of the Cap‘n’Collar transcription factors. Cell. Mol. Life Sci..

[37] Sankaranarayanan K., Jaiswal A.K. (2004). Nrf3 negatively regulates antioxidant-response element-mediated expression and antioxidant induction of NAD(P)H:quinone oxidoreductase1 gene. J. Biol. Chem..

[38] Merry T.L., Ristow M. (2016). Nuclear factor erythroid-derived 2-like 2 (NFE2L2, Nrf2) mediates exercise-induced mitochondrial biogenesis and the anti-oxidant response in mice. J. Physiol..

[39] Piantadosi C.A., Carraway M.S., Babiker A., Suliman H.B. (2008). Heme oxygenase-1 regulates cardiac mitochondrial biogenesis via Nrf2-mediated transcriptional control of nuclear respiratory factor-1. Circ. Res..

[40] Dinkova-Kostova A.T., Abramov A.Y. (2015). The emerging role of Nrf2 in mitochondrial function. Free Radic. Biol. Med..

[41] Kovac S., Angelova P.R., Holmström K.M., Zhang Y., Dinkova-Kostova A.T., Abramov A.Y. (2015). Nrf2 regulates ROS production by mitochondria and NADPH oxidase. Biochim. Biophys. Acta.

[42] Holmström K.M., Baird L., Zhang Y., Hargreaves I., Chalasani A., Land J.M., Stanyer L., Yamamoto M., Dinkova-Kostova A.T., Abramov A.Y. (2013). Nrf2 impacts cellular bioenergetics by controlling substrate availability for mitochondrial respiration. Biol. Open.

[43] Wadhwa R., Taira K., Kaul S.C. (2002). An Hsp70 family chaperone, mortalin/mthsp70/PBP74/Grp75: What, when, and where?. Cell Stress Chaperones.

[44] Zhang Y., Ahn Y.H., Benjamin I.J., Honda T., Hicks R.J., Calabrese V., Cole P.A., Dinkova-Kostova A.T. (2011). HSF1-Dependent Upregulation of Hsp70 by Sulfhydryl-Reactive Inducers of the KEAP1/NRF2/ARE Pathway. Chem. Biol..

[45] McGarry J.D., Foster D.W. (1980). Regulation of hepatic fatty acid oxidation and ketone body production. Annu. Rev. Biochem..

[46] Newman J.C., Verdin E. (2014). Ketone bodies as signaling metabolites. Trends Endocrinol. Metab..

[47] Cotter D.G., Schugar R.C., Crawford P.A. (2013). Ketone body metabolism and cardiovascular disease. Am. J. Physiol. Heart Circ. Physiol..

[48] Rotondo F., Ho-Palma A.C., Remesar X., Fernández-López J.A., del Mar Romero M., Alemany M. (2017). Glycerol is synthesized and secreted by adipocytes to dispose of excess glucose, via glycerogenesis and increased acyl-glycerol turnover. Sci. Rep..

[49] Rudich A., Tirosh A., Potashnik R., Hemi R., Kannety H., Bashan N. (1998). Prolonged oxidative stress impairs insulin-induced GLUT4 translocation in 3T3-L1 adipocytes. Diabetes.

[50] Saleh S., El-Ridi M., Zalat S., El-Kotb S., Donia S. (2014). Additive effect of ozone therapy to insulin in the treatment of diabetic rats. Menoufia Med. J..

[51] Pi J., Leung L., Xue P., Wang W., Hou Y., Liu D., Yehuda-Shnaidman E., Lee C., Lau J., Kurtz T.W. (2010). Deficiency in the nuclear factor E2-related factor-2 transcription factor results in impaired adipogenesis and protects against diet-induced obesity. J. Biol. Chem..

[52] Schneider K.S., Chan J.Y. (2013). Emerging role of Nrf2 in adipocytes and adipose biology. Adv. Nutr..

[53] Zhang Z., Zhou S., Jiang X., Wang Y.H., Li F., Wang Y.G., Zheng Y., Cai L. (2015). The role of the Nrf2/Keap1 pathway in obesity and metabolic syndrome. Rev. Endocr. Metab. Disord..

[54] Kim B.R., Lee G.Y., Yu H., Maeng H.J., Oh T.J., Kim K.M., Moon J.H., Lim S., Jang H.C., Choi S.H. (2018). Suppression of Nrf2 attenuates adipogenesis and decreases FGF21 expression through PPAR gamma in 3T3-L1 cells. Biochem. Biophys. Res. Commun..

[55] Yoo H., Antoniewicz M.R., Stephanopoulos G., Kelleher J.K. (2008). Quantifying reductive carboxylation flux of glutamine to lipid in a brown adipocyte cell line. J. Biol. Chem..

[56] Eliasen M.M., Brabec M., Gerner C., Pollheimer J., Auer H., Zellner M., Weingartmann G., Garo F., Roth E., Oehler R. (2006). Reduced stress tolerance of glutamine-deprived human monocytic cells is associated with selective down-regulation of Hsp70 by decreased mRNA stability. J. Mol. Med..

[57] Bocci V., Aldinucci C., Mosci F., Carraro F., Valacchi G. (2007). Ozonation of human blood induces a remarkable upregulation of heme oxygenase-1 and heat stress protein-70. Mediators Inflamm..

[58] Niki E. (2016). Oxidative stress and antioxidants: Distress or eustress?. Arch. Biochem. Biophys..

[59] Chusyd E.D., Wang D., Huffman D.M., Nagy T.R. (2016). Relationships between rodent white adipose fat pads and human white adipose fat depots. Front. Nutr..

