# Supplementary material for: Ozone Activates the Nrf2 Pathway and Improves Preservation of Explanted Adipose Tissue In Vitro
Source: Antioxidants (Basel). 2020 Oct 14;9(10):989. doi: 10.3390/antiox9100989 (PMC7602229; doi:10.3390/antiox9100989)
Supplement: Supplementary file 1 [file antioxidants-09-00989-s001.pdf]

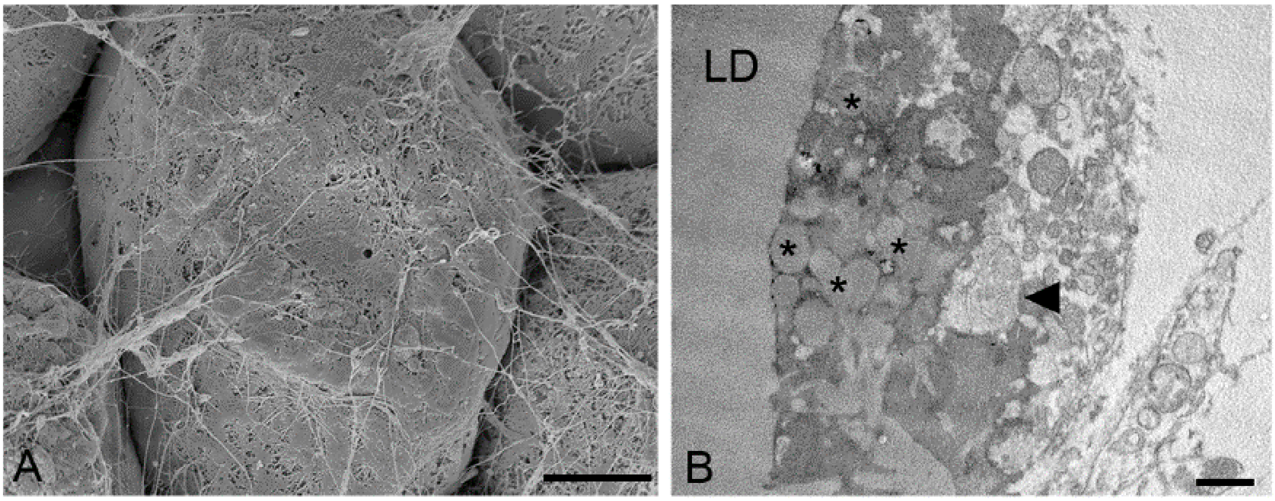

Figure S1. SEM (A) and TEM (B) micrographs of adipocytes after 2 h from treatment with 100 μg O_3. Adipocytes are wrinkled (A) and seriously injured (B), showing massive loss of lipid droplets (asterisks) and damaged organelles (note the hardly recognizable mitochondria, arrowhead). Bars, 10 μm (A), 500 nm (B).

Table S1. Coefficient of variation (CV% = standard deviation / mean x 100) for each metabolite analyzed (n=2).

Metabolite		2 h	24 h	48 h
3-hydroxybutyrate	control	1.06	8.22	2.10
	O_2	6.56	4.41	2.58
	10 μg O_3	5.49	2.00	0.41
	20 μg O_3	3.37	0.64	2.12
isovaleric acid	control	1.78	3.97	0.10
	O_2	1.54	2.70	0.63
	10 μg O_3	0.30	0.50	0.50
	20 μg O_3	0.53	6.10	0.53
glucose	control	0.69	0.17	0.42
	O_2	0.74	0.09	2.87
	10 μg O_3	2.37	0.42	2.27
	20 μg O_3	1.04	1.07	1.53
glutamine	control	0.19	0.27	14.22
	O_2	0.80	1.76	11.46
	10 μg O_3	0.40	0.33	0.32
	20 μg O_3	0.42	0.13	4.62
glycerol	control	0.49	0.57	3.36
	O_2	0.31	0.32	8.82
	10 μg O_3	0.46	0.02	0.53
	20 μg O_3	0.36	0.60	1.75

Multivariate data analysis

Methods

For data processing and multivariate analysis, an exponential line broadening (0.3 Hz) was applied to FIDs before Fourier transform. The spectra were manually phased, baseline corrected and aligned with reference to the TSP signal at δ 0.00 ppm. Each spectrum in the region 9.00-0.1 ppm was segmented into identical intervals of 0.03 ppm (bins) and the signal intensity in each interval was integrated, excluding the region that contains the residual water peak. The integrals were normalized with respect to their sum and then statistically evaluated using Metaboanalyst software (www.metaboanalyst.ca). Principal component analysis (PCA) was used to assess the presence of initial group clustering between the data set and to identify the outliers. Partial least squares discriminant analysis (PLS-DA) was used to further maximize the discrimination between groups and to determine the metabolites that contributed to the variation. The significant identified NMR signals were assigned according to the assignments published in the literature and by comparison with spectra of standard compounds deposited in databases (Biological Magnetic Resonance Data Bank, BMRB, http://www.bmrb.wisc.edu/ and Human Metabolome Data Base, HMDB, http://www.hmdb.ca/) and with the aim of the web Bayesil system.

Results

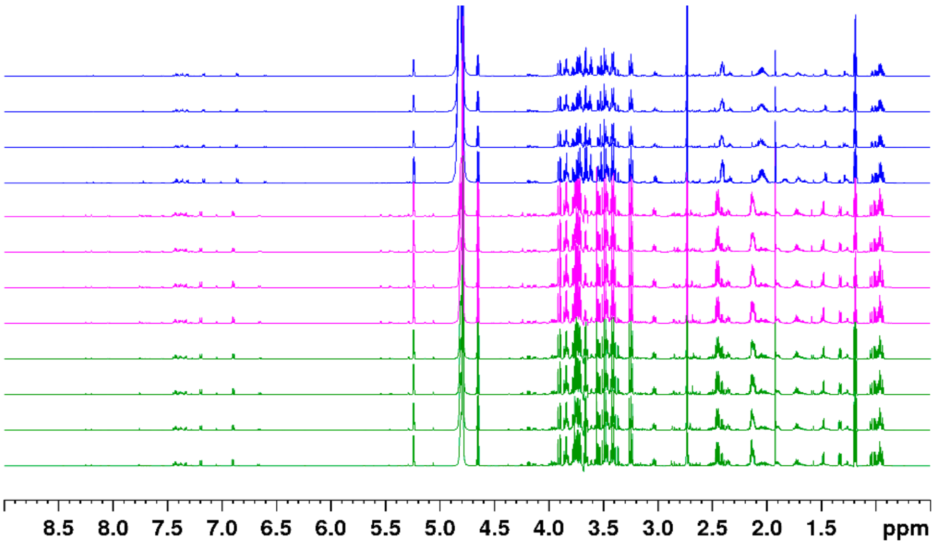

Figure S2. Representative NMR spectra recorded on culture media after 2 h (green), 24 h (magenta) and 48 h (blue) from gas treatment.

PCA was initially performed to generate an overview of trends, grouping, and potential outliers in the ^{1}H NMR data set. As shown in Figure S3A, the PCA score plot indicates a cluster separation of the media samples along the time. In order to maximize the difference among groups and identify the significant loadings, we carried out the PLS-DA analysis. Figure S3B displays the PLS-DA 2D scores plot which demonstrates a clear separation of the samples collected at different times. The quality of the OPLS-DA models was estimated by 10-fold cross validation using R2 and Q2 values, which refer to the goodness of fit and the quality of prediction, respectively. Our model scored an accuracy=1, R^2=0.96 and Q^2=0.82, suggesting that the model was a good fit. The most influential peaks in the models were identified and a metabolite assigned to each peak.

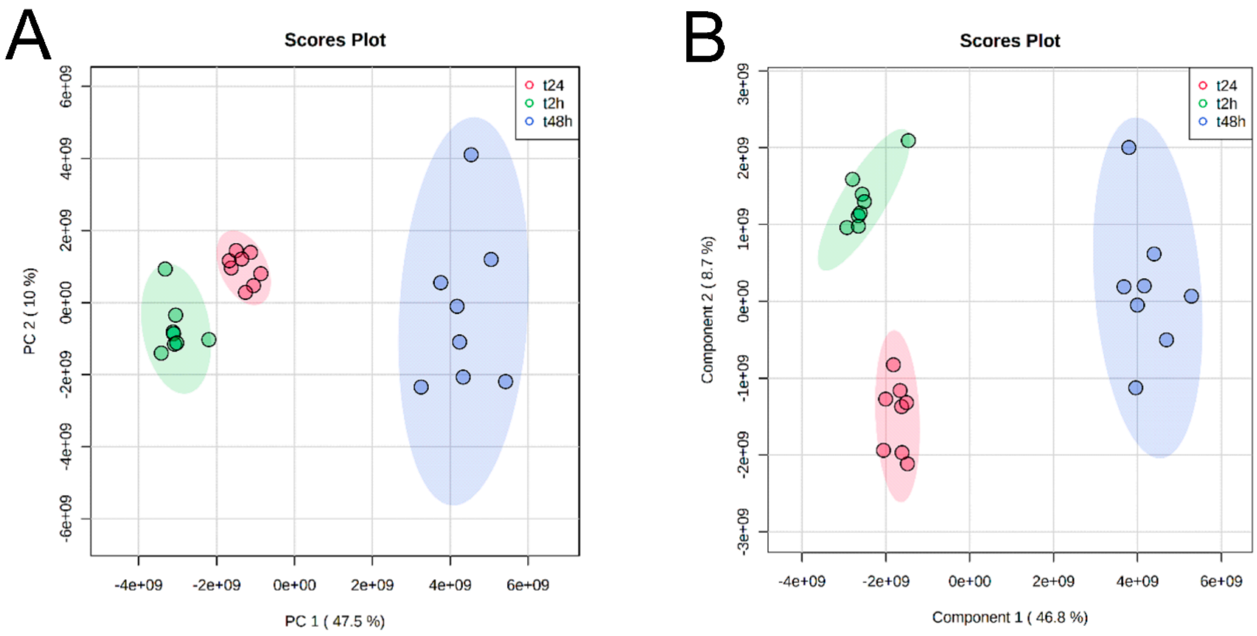

Figure S3. NMR-based metabolomic analysis of culture medium samples. PCA (A) and PLS-DA (B) score plots derived from 1H-NMR spectra data.
